# Trauma, Temperament, Alexithymia, and Dissociation Among Persons Addicted to Alcohol: Mediation Model of Dependencies

**DOI:** 10.3389/fpsyg.2018.01570

**Published:** 2018-09-07

**Authors:** Elżbieta Zdankiewicz-Ścigała, Dawid K. Ścigała

**Affiliations:** ^1^Faculty of Psychology, SWPS University of Social Sciences and Humanities, Warsaw, Poland; ^2^Institute of Psychology, Faculty of Applied Social Sciences, The Maria Grzegorzewska University, Warsaw, Poland

**Keywords:** alexithymia, dissociation, temperament, trauma, addiction

## Abstract

**Objective:** The attachment theory has been conceptualized as an affect regulation theory, proposing that attachment is associated with the expression and recognition of emotions as well as interpersonal functioning. The purpose of the study was to examine a model, in which a relation was analyzed between childhood trauma, temperament, alexithymia, and dissociation in a group of individuals addicted to alcohol.

**Method:** The total number of 201 persons were examined, comprising 67 women (33.3% of participants) and 134 men (66.7% of participants). The participants aged from 18 to 68 (*M* = 32.81; *SD* = 12.12). In order to measure the analyzed variables, the following questionnaires were applied: MAST, SSSV, TAS20, TEC, and CES.

**Results:** A comparative analysis between the group of alcohol addicts and non-addicts showed statistically significant differences related to: the intensity of trauma, temperament, alexithymia, and dissociation. The study of models related to the impact of traumatic experience intensity on the level of alcohol addiction with regard to a mediatory role of alexithymia, dissociation, and temperament showed the existence of several important indirect effects, and the model, which takes into account all three mediators, is statistically significant *F*(4,196) = 35.1964; *p* < 0.001.

**Conclusion:** Childhood trauma, as well as alexithymia and dissociation block dealing with stress based on self-reflection and self-control, and contribute to affective disorders and their regulation with alcohol.

**Limitations:** The selection of participants to homogeneous groups with regard to age and gender constituted the most important difficulty and limitation. A perfect age criterion for investigating the interaction between the temperament and the consequences of traumatic development would be early adulthood.

## Introduction

The attachment theory has been conceptualized as an affect regulation theory, proposing that attachment is associated with the expression and recognition of emotions as well as interpersonal functioning (Bowlby, 1988). From a developmental – organismic perspective early experience has special significance because it provides the foundation on which all subsequent adaptations are constructed (Sroufe, 2005). Like the foundation of a building on which a range of structures may be built, early experience does not determine a specific course of adaptive functioning, but rather constrains its form in a probabilistic fashion. The crux of the developmental position is that both competence and maladaptation arise from the same transactional and cumulative processes over time (Yates et al., 2011). A child growing up in a cold, distant and emotionally negligent atmosphere might have difficulties developing emotional abilities such as self-awareness and introspection to the same extent as a child growing up in a warm, protective, and emotionally supportive home (Thompson, 2008; Yates et al., 2011). This form of emotional maltreatment can impede a secure attachment between caregiver and care recipient (Bekker et al., 2007; Grusec, 2011) and might result in the development of altered emotional abilities (Montebarocci et al., 2004; Frewen et al., 2008). Early trauma disturbs the development of cognitive and affective processing, the integration of thinking and feeling, as well as the capacity. The inability to identify or name emotions, coupled with the feeling of being overwhelmed, may cause individuals to experience trauma. These individuals may become accustomed to expressing their affect (van der Kolk and McFarlane, 1996; Zdankiewicz-Ścigała, 2017) through the development of a compensatory, non-verbal strategy, such as pathological drinking, to disrupt a sense of psychological numbing and/or to avoid and manage intense, seemingly uncontrollable emotions to understand and express emotional states (Yates, 2009). These disturbances are linked to post-traumatic reactions, dissociation, and alexithymia (Craparo et al., 2014; Zdankiewicz-Ścigała, 2017).

The relations between childhood traumatic experiences, and dissociation and alexithymia with reference to persons addicted to alcohol formed the basis for the study carried out by Craparo et al. (2014). The purpose of the study was to verify the assumptions regarding the relation between the type of childhood traumatic experience, temperament and dissociation and alexithymia, in alcohol addiction. The conducted analysis revealed more frequent occurrence of traumatic experiences in a clinical group compared to a control group, in particular, emotional and physical violence. Alcohol addicts experienced traumatic events in their young age. In addition, a higher score for dissociation and alexithymia was stated in a group of addicts. In a study carried out by Zdankiewicz-Ścigała (2017), a theoretical model was examined, in which connections between attachment styles, trauma, and alexithymia and dissociation were investigated in persons addicted to alcohol. The theoretical model subject to final empirical verification enabled the identification of mechanisms which form the basis for affective development disorders, examined based on the example of alcohol addiction. With structural equation models (i.e., AMOS, GLS), the adjustment of theoretical model to data was examined, which allowed the description of dependency paths. The strongest relation was proven in the case of an evasive attachment style, which turned out a strong predictor fostering the development of alexithymia and the occurrence of traumas related to emotional negligence and mental violence. The carried out analyses enabled to conclude that traumatic experiences and alexithymia foster the development of an inclination to pathological dissociation, and impact directly on the deregulation of affective processes. No direct impact of dissociation on the development of addiction was proven. The impact of attachment styles and dissociation was of an indirect nature, which does not change the fact that it is the disorganized attachment that determines the development of dissociation, and fosters the occurrence of trauma. On the one hand, trauma is a source of disorganized attachment; on the other hand this attachment style may foster risky behaviors which increase the probability of revictimization (Schore, 2009).

Thorberg et al. (2009) have demonstrated that alexithymia is perceived as the main predictor of addiction to alcohol, and its share is estimated between 45 and 67%. Whereas the origin of alexithymia as a personality trait (Messina et al., 2014) is to be related to emotional negligence resulting from non-secure bonds of attachment and development trauma, the deficits may be considered as a very important factor, which not only fosters disorders in the development of capabilities to understand and express emotions (Craparo et al., 2014), but also contributes to decompensation on the neurophysiological level (Schore, 2000, 2009). Trauma experienced by a child in the form of emotional negligence, or emotional violence, due to the fact that it evokes chronic agitation condition, requires the experiencing person (a child) to develop a strategy of dealing with the negative emotions. The most frequently applied strategies are a disconnection from emotional feelings and a failure to communicate experienced feelings to others. The two strategies may constitute the basis for the development of alexithymia (Sifneos, 1991), and the inclination to pathological dissociation (Liotti, 2004), among others. The term alexithymia is used to describe problems related to the cognitive processing of affectional stimulation, and thereby deficits in regulating emotions and understanding physiological correlates of emotions. The deficits relate to difficulties in identifying and verbalizing emotions, and refer to thinking which only focuses on external factors evoking emotional arousal, i.e., operational style of thinking (Taylor et al., 1990). On the other side, dissociation is defined as splitting normally integrated functions of awareness, memory, and identity. It is a mechanism which blocks the integration of threatening content in the awareness, and gets activated exclusively in circumstances in which an individual does not have a chance to process impulse stimulation in other manner. This may adopt the form of detachment and compartmentalization of experience (Liotti and Farina, 2016). The more anxiety related attachment, the stronger affective dysregulation, and the higher risk of exposure to traumas, both the relational ones (including small “t” infidelity traumas) (Freyd, 1994), as well as capital “T” traumas (Liotti, 2004). As Schore (2000) indicates, as a result of persistent stress in which a child is brought up, the inefficient regulation of ANS is carried out by higher centers in the CNS, which is reflected by the disturbance of central regulation of sympathetic nervous system, as well as the hypothalamus – pituitary gland – adrenal cortex axis. According to the scholar, the loss of such regulation means that in a situation of stress, the mechanism of combined mutual autonomous control gives way to the mechanism of combined non-mutual autonomous control, as a result of which the extremely high arousal of both sympathetic nervous system and parasympathetic nervous system is experienced (Schore, 2009). Traumatic experiences at young age “excite the brain’s limbic system,” leaving the permanent physiological responsiveness in this system, and restraining the capability of coping with stressors in the future (Allen et al., 2014). The episodes of post-traumatic disorders in infancy, according to Schore (2009), in the form of excessive agitation and dissociation, are subject to imprinting, constituting the pattern for further post-traumatic disorders in the childhood, adolescence, and adulthood. In all abovementioned disorders, the following are stated: the disturbances of autonomic system’s reactions to agitation, the dysfunctions of catecholamines’ release, mild neurological symptoms, and dissociation (Schore, 2000). The final effect of experiencing permanent distress evoked by traumatic relation in the early period of life is the progressive decline in an ability to adjust flexibly, undertake defensive actions, and act in line with one’s own interest, as well as blocking the capability to register emotions and pain (Schore, 2000, 2009; Allen et al., 2014). Anxiety related attachment styles and traumatic experiences are not conductive to the appropriate development of the brain, neither as regards structures or relations among them, nor as regards functions (Schore, 2009). This pathology relates to the “disintegration” or decompensation under stress, which means the loss of an ability to restrain agitation by the evolutionarily latest layers of the nervous system, related to higher functions, and the release of lower, more autonomous functions. In extreme stress conditions, in the absence of correctly operating brain systems, the loss of orbitofrontal cortex functions takes place through the loss of anterior cingulate cortex functions, and the last element is the dysfunction of amygdala (Schore, 2009).

From the point of biology, the vulnerability to regulating emotions with stimulants (including alcohol) depends on the features of temperament. The sensations seeking is, according to Zuckerman (2007), the need to experience new, complex feelings and experiences, as well as the readiness to undertake the social and physical risk in order to provide oneself with this type of experiences. A positive emotional condition arises when such experiences are provided, which stimulate the nervous system optimally. The research carried out on animals shows that the limbic system and monoamine systems control behaviors, which Zuckerman calls “sensation seeking” (Strelau, 2002). Zuckerman’s studies indicate that sensation seeking depends on the level of catecholamines, i.e., adrenaline, noradrenaline, and dopamine in the limbic system areas, and on the level of platelet monoamine oxidase (MAO). MAO enzyme blocks the activity of catecholamines by decreasing their level, and as a result, it leads to the fall of behavior activity. Zuckerman treats MAO enzyme as an inhibitor of sensation seeking, and indicates the negative dependency between those two variables (Zuckerman, 2007). As another study shows (Darkes et al., 1998; Crawford et al., 2003), the sensation seeking feature enables forecasting the development of alcohol abuse from the early adolescence. Persons who reach for alcohol in adolescence will not develop this inclination in a period of later adulthood, to such a great extent, as persons characterized by the high level of sensation seeking. It is worth mentioning that the peak period for the development of risky behaviors, such as the development of alcohol addictions is a period of early adulthood. This is due to the developmental asymmetry, i.e., reaching the early peak of sensation seeking feature combined with an undeveloped mechanism of emotions modulation (McCabe et al., 2015). According to those authors’ study results, persons characterized by the high level of sensation seeking and the low level of emotions’ modulation reached the highest level of alcohol abuse. Therefore, a need for stimulation in early adulthood and the affection regulation dysfunction in the form of alexithymia should be considered as a key risk factor for the development of addictions, as well as the development of their correlated mental disorders.

Studies carried out by Evren and Evren (2006) on the relation of negligence and emotional violence with the intensity of alexithymia in men addicted to alcohol and after suicide attempts at the same time, revealed that the higher the intensity of traumas, the higher the level of alexithymia in relation to difficulties in identifying and verbalizing emotions. Apart from those variables, in a group of men after suicide attempts, lower scores were shown in the scale of self-direction and the scale of willingness to cooperate, pursuant to Cloninger’s concept (Cloninger, 1997). In the case of other factors, no significant differences were shown. The results of hitherto studies did not take into account all factors which foster the development of addictions simultaneously.

The purpose of the study is to verify how alexithymia, dissociation, and temperament mediate a relation between the impact of trauma intensity and the level of alcohol addiction. Both alexithymia and dissociation may constitute factors which influence the process of mental stress accumulation, and constitute, with the involvement of temperament, the basis for the release of tension in an impulsive manner with psychoactive substances, among others. Impulsive and compulsive behaviors observed in persons, who are addicted to alcohol, constitute the pathological expression of an individual, who is forced to act, basing on the anxiety and dysphoric state developed based on the relation trauma in a childhood period. Such behaviors aim to create self-calming mental shelters: they are shaped in the form of certain sensory experiences and altered states of consciousness, which are first used to defend, but they further lead an individual to live in a constant condition of absorption and dissociation, which leads to addiction (Craparo et al., 2014).

## Materials and Methods

This study was carried out in accordance with the recommendations of the SWPS University of Social Sciences and Humanities Ethics Committee with written informed consent from all participants. All procedures performed in studies involving human participants were in accordance with the ethical standards of the institutional and/or national research committee and with the 1964 Helsinki declaration and its later amendments or comparable ethical standards. An ethics approval for this research was not required as per the SWPS University of Social Sciences and Humanities Ethics Committee’s guidelines and national regulations.

In the procedure of this research there was used only standardized questionnaires, which are used in psychological worldwide research – this type of research is based on guidelines and procedures in accordance with applicable law and ethics, but does not require individual consent. Consent to the study was approved by the appropriate authorities of the therapeutic departments and the patients themselves. It was carried out in addiction therapy departments by psychologists working permanently with patients. Before starting to fill in the questionnaires, they were asked to sign an informed consent form which included all their tasks and rights. The total number of 201 persons were examined from three addiction treatment centers from the wards for group therapy of alcohol addiction, comprising 67 women (33.3% of participants) and 134 men (66.7% of participants). The participants aged from 18 to 68 (*M* = 32.81; *SD* = 12.12). Based on a MAST questionnaire for diagnosing alcohol addiction, participants were divided into a control group (a score below 4 points), a group of likely addicted individuals (a score of 4 points), and a group addicted individuals (a score of 5 points and over). The control group comprised 100 individuals (49.8% of participants), 40 women and 60 men, at the age of 18 to 50 (*M* = 25.50; *SD* = 7.40). The group of likely addicted individuals comprised 4 persons (2.0% of participants), exclusively men at the age of 21 to 27 (*M* = 23.25; *SD* = 2.63). The alcohol addicts group comprised 97 persons (48.3% of participants), 27 women and 70 men at the age of 20 to 68 (*M* = 40.73; *SD* = 11.28). All persons, who were patients of addiction treatment centers, entered the group of alcohol addicts.

## Measures

### Trauma

The Traumatic Experiences Checklist (TEC). TEC (Nijenhuis et al., 2002) was applied to investigate the intensity of traumatic experiences. In the study, a general scale of traumatic experiences was used exclusively, but the questionnaire also enables the calculation of scores for particular trauma categories. A participant responds to 29 questions which refer to potentially traumatic events. For each question, an individual is required to provide information whether a given event took place in the life of responding person; the age of person when the trauma was suffered; the duration and the subjective level of trauma’s impact on the life of the individual (choice within the range of 1 to 5). The more points, the greater intensity of traumatic experiences. Cronbach’s α coefficient on the level of 0.91 means the good reliability of scale, and studies which compare it to SLESQ – Stressful Life Experiences Questionnaire, prove high accuracy (*r* = 0.77; *p* < 0.0001) (Nijenhuis et al., 2002).

### Alexithymia

The Toronto Alexithymia Scale *– 20 (TAS-20).* TAS-20 (Parker et al., 1993) was applied to investigate the level of alexithymia. Apart from the general level of alexithymia, the questionnaire allows for estimating separate scales for dimensions such as: “difficulties in verbalizing feelings”; “difficulties in identifying feelings”; “operational style of thinking.” The questionnaire comprises 20 test items. Each item has a five-degree Likert scale (1-strongly disagree; 2-partially disagree; 3-undecided; 4-partially agree; 5-strongly agree). The scale is from 20 to 100 points. It is a reliable and accurate tool. In relation to the Polish version, Cronbach’s α coefficient is 0.73 for the general score; 0.55 for the “difficulties in verbalizing feelings” scale; 0.71 for the “difficulties in identifying feelings” scale; and 0.51 for the “operational style of thinking” scale.

### Dissociation

The Curious Experiences Survey (CES). CES (Goldberg, 1999) was applied to investigate a tendency toward dissociation. The survey enables estimating the general level of tendency toward dissociation and the scales included: “amnesia,” “absorption,” “depersonalization.” The survey comprises 31 test items. Each item has a five-degree Likert scale (1-it never happens to me; 2-it rarely happens to me; 3-it sometimes happens to me; 4-it often happens to me; 5-it always happens to me). The scale is from 31 to 155 points. Psychometric tests carried out for the original language version indicate that it is a reliable tool; Cronbach’s α coefficient for calculating reliability is 0.91 for the general scale; 0.75 for “amnesia”; 0.76 for “absorption,” and 0.88 for “depersonalization” scale.

The Sensation Seeking Scale – V (SSS-V). SSS-V (Zuckerman et al., 1978) was applied to investigate the level of sensation seeking and the intensity of individual dimensions of this feature. The tool enables estimating a general level of sensation seeking and 4 covered scales: “thrill and adventure seeking,” “experience seeking,” “disinhibition,” “boredom susceptibility.” SSS-V comprises 40 test items, which include two statements each (“a” and “b”). One point is given for each response in line with the key. The subject may be awarded from 0 to 40 points in a general score, and from 0 to 10 points on each individual scale. Survey results in relation to the original language version confirm relevant psychometric properties of this tool (Roberti et al., 2003). Cronbach’s α coefficient is 0.80 for “thrill and adventure seeking”; 0.75 for “experience seeking”; 0.80 for “disinhibition”; 0.76 “boredom susceptibility” scale.

### Addiction to Alcohol

The Michigan Alcoholism Screening Test (MAST). MAST (Selzer, 1971) was applied to investigate the intensification of alcoholic behaviors. The questionnaire is a screening test; it comprises 24 questions to which a subject responds “yes” or “no.” Questions are valued 0 to 5 points. The score on a general scale is from 0 to 53 points. Obtaining 5 or more points means the statement of alcoholism, according to DSM-IV-TR classification. MAST provides results of sufficient reliability for research purposes, but considerable caution is advised when applying the one (Shields et al., 2007). As studies show, results of MAST are less reliable in the case of women and non-clinical cases, however, applying this tool in a clinical group, as a quantitative indicator of alcohol addiction, is fully justified.

## Results

The statistical analysis, which allowed testing formulated hypotheses, was carried out in IBM SPSS Statistics program, release 24. The program was used to analyze basic descriptive statistics, due to which it was possible to examine the distribution of subsequent measured variables. The hypotheses were tested using a series of correlation analyses, analyses of variances, and analyses mediations using PROCESS macro by Hayes (2013). A typical threshold, i.e., α = 0.05 was the adopted significance level. In the examined group of alcohol addicts, the mental negligence occurred in 56% of addicted, mental violence in 44%, physical violence in 43.6%, physical punishment in 41%, sexual harassment in 7.8%, and sexual abuse in 8.3%. The strength of those experiences is significantly higher than in a group of non-addicts. Respectively: 23, 26, 18, 29.1, 3.2, and 2.1%. The intensity level of traumatic experiences in the whole group amounted to *M* = 5.63; *SD* = 3.82. The level of alexithymia in the examined group amounted to *M* = 51.29; *SD* = 14.00. The scores for individual scales were as follows: difficulties in verbalizing emotions *M* = 14.58; *SD* = 4.72; difficulties in identifying emotions *M* = 18.50; *SD* = 6.61; the operational style of thinking *M* = 18.21; *SD* = 4.79. Dissociation general score amounted to *M* = 53.26; *SD* = 14.86; amnesia *M* = 11.83; *SD* = 3.70; absorption *M* = 25.95; *SD* = 7.78; depersonalization *M* = 15.49; *SD* = 5.36. Sensation seeking general score amounted to *M* = 18.23; *SD* = 5.98; thrill and adventure seeking *M* = 5.33; *SD* = 2.81; internal experience seeking *M* = 5.59; *SD* = 1.92, disinhibition *M* = 4.29; *SD* = 2.42; boredom susceptibility *M* = 3.02; *SD* = 1.93. Before more advanced statistical analyses were started, the analyses were carried out with an aim to verify whether statistically significant differences exist between the group of alcohol addicts and the control group as far as alexithymia, dissociation, experience seeking, and intensity of traumatic experience levels are concerned. For this purpose, in relation to alexithymia level, a single factor analysis of variance (ANOVA) was carried out in an inter-group schema. The analysis revealed significant differences in the general level of alexithymia; *F*(1,195) = 51.505; *p* < 0.001 and in sub-scales: difficulty in identifying feelings *F*(1,195) = 43.824; *p* < 0.001; difficulty in verbalizing feelings *F*(1,195) = 36.671; *p* < 0.001; and in relation to the operational style of thinking *F*(1,195) = 27.589; *p* < 0.001). A similar analysis was performed for dissociation. The significant differences were revealed for the general level of dissociation intensity; *F*(1,195) = 60.051; *p* < 0.001 and in relation to sub-scales: absorption *F*(1,195) = 33.163; *p* < 0.001, depersonalization *F*(1,195) = 54.601; *p* < 0.001, amnesia *F*(1,195) = 55.267; *p* < 0.001). The mean score for non-addicts amounted to *M* = 46.02; *SD* = 10.10, and for addicts it amounted to *M* = 60.23; *SD* = 15.23. Persons addicted to alcohol showed the significantly higher level of traumas intensity (*M* = 5.63; *SD* = 3.82) than individuals who were not addicted to alcohol (*M* = 2.98; *SD* = 1.97); *F*(1,195) = 24.585; *p* < 0.001). A significant difference was revealed as a result of the performed analysis as regards the general level of sensation seeking, which for the group of alcohol addicts amounted to (*M* = 19.38; *SD* = 6.5), and for non-addicts it amounted to (*M* = 17.00; *SD* = 5.18); *F*(1,195) = 8.104; *p* < 0.05. In addition, a significantly higher result was obtained as regards disinhibition among addicted individuals (*M* = 5.49; *SD* = 2.45), the non-addicted (*M* = 3.18; *SD* = 1.82); *F*(1,195) = 56.471; *p* < 0.001. In the next stage, the correlation analyses were carried out between independent variables and the main dependent variable, i.e., an addiction to alcohol. *Pearson’s r* correlation analysis was performed.

The performed *Pearson’s r* analysis revealed a positive strong relation *r* = 0.514; *p* < 0.001, which shows that the higher intensity of traumatic experiences, the higher inclination toward addiction. In order to verify the mediation role of alexithymia, the analysis of mediations was carried out, supplemented by the Sobel test using a non-standard macro for Hayes’ SPSS Process (2013). The results of analysis confirmed the mediation role of alexithymia in the relation between the intensity of traumatic experiences and alcohol addiction *z* = 3.935; *p* < 0.001. The mediation is partial. The strength of a relation between an independent variable and a dependent variable after the introduction of a mediator decreased from *c* = 0.51; *p* < 0.001 to *c*′ = 0.39; *p* < 0.001, but it was still significant. It is worth mentioning that the mediation model explains 36% of alcohol addiction scores’ variability.

**FIGURE 1:**
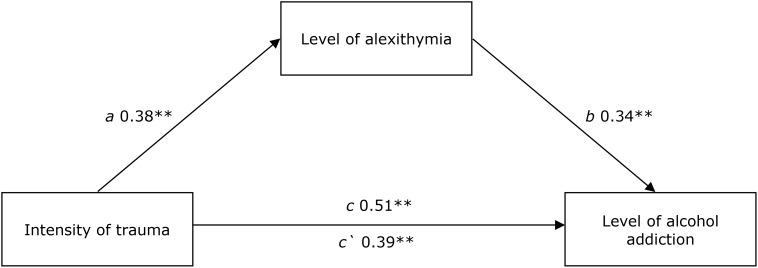
The model of mediation for the impact of trauma intensity alexithymia level on an inclination toward alcohol addiction with the mediation impact of alexithymia. ^∗^*p* < 0.05; ^∗∗^*p* < 0.01. Direct effect – X on Y without the influence of M1 – c. Indirect effect of X on Y through M1 – a, b. Direct effect – X on Y including the influence of M1 – c‘.

**Figure 1** presents the obtained standardized beta coefficients.

**FIGURE 2:**
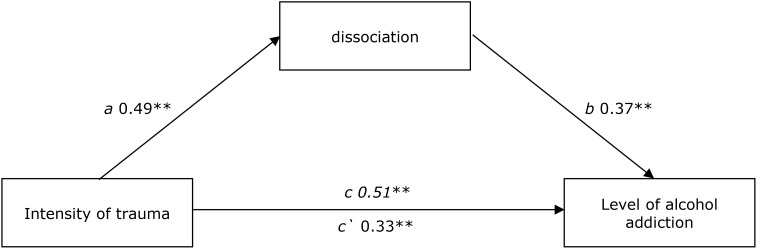
The model of mediation for the tendency toward dissociation between the impact of trauma intensity on the level of alcohol addiction. ^∗^*p* < 0.05; ^∗∗^*p* < 0.01. Direct effect – X on Y without the influence of M1 – c. Indirect effect of X on Y through M1 – a, b. Direct effect – X on Y including the influence of M1 – c‘.

Another analysis, which was performed, related to the specification of dissociation’s role as a mediator between trauma and alcohol addiction. The assumption was confirmed of the mediation role of dissociation between the intensity of traumatic experiences and alcohol addiction *z* = 4.61; *p* < 0.001. The mediation is also of a partial nature. **Figure 2** presents the obtained standardized beta coefficients.

Disinhibition turned out to be the only dimension of sensation seeking, which is moderately strongly related to an inclination toward alcohol addiction. The obtained score formed the basis for performing a stepwise regression analysis, in which the following factors constituted inclination forecasting predictors: disinhibition, dissociation, and alexithymia. The model, which took account of three predictors, turned out to be well fitted to data *F*(3,147) = 40.603738; *p* < 0.001, as explained 44% of dependent variable’s variances. Disinhibition (β = 0.391; *p* < 0.001) proved to be the strongest predictor in the model; dissociation (β = 0.275; *p* < 0.001) and alexithymia (β = 0.232; *p* < 0.001) involved a dependent variable to a similar extent. In order to estimate the dependency of dissociation and disinhibition, as well as their interactions with the inclination toward alcohol, the regression analysis was performed with an interactive component. Independent variables were aligned with the standardization of results. Dissociation (β = 0.382; *p* < 0.001) and disinhibition (β = 0.427; *p* < 0.001) were significant predictors, and the introduced introduction component (β = -0.0658; *p* > 0.05) turned out statistically insignificant, and it did not improve the predictive value of the model *F*(3,147) = 35.216; *p* < 0.001. In order to examine the dependency of alexithymia and disinhibition, as well as their interactions with the inclination toward alcohol, the regression analysis was performed with an interactive component. Independent variables were aligned with the standardization of results. The performed hierarchical regression analysis with an interactive component confirmed the significance of alexithymia (β = 0.367; *p* < 0.001), disinhibition (β = 0.459; *p* < 0.001), and the interactive component (β = -0.148; *p* < 0.03) *F*(3,147) = 35.216; *p* < 0.001. The introduction of an interactive component improved the per cent value of a dependent variable’s explanation by 2%.

**FIGURE 3:**
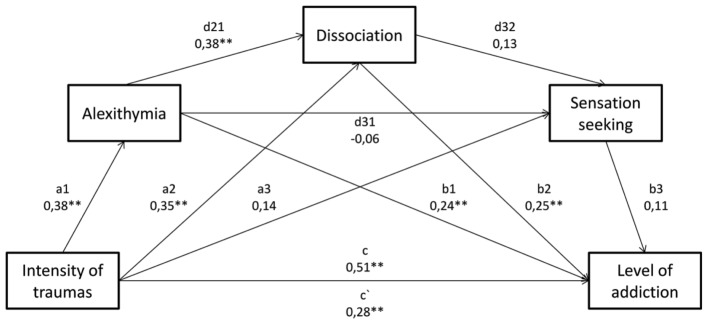
The model of mediation for the impact of traumatic experiences’ intensity on the inclination toward alcohol addiction, with three mediators, i.e.: alexithymia, dissociation, and sensation seeking. Below, intermediate effects were described, which were calculated in the model. ^∗^*p* < 0.05; ^∗∗^*p* < 0.01. Direct effect – X on Y without the influence of M1, M2 or M3 – c. Effect 1 – Trauma – > Alexithymia – > Addiction – significant a1, b1. Effect 2 – Trauma – > Alexithymia – > Dissociation – > Addiction – significant a1, d21, b2. Effect 3 – Trauma – > Alexithymia – > Sensation seeking – > Addiction – N.I. a1, d31, b3. Effect 4 – Trauma – > Alexithymia – > Dissociation – > Sensation seeking – > Addiction – N.I. a1, d21, d32, b3. Effect 5 – Trauma – > Dissociation – > Addiction – significant a2, b2. Effect 6 – Trauma – > Dissociation – > Sensation seeking – > Addiction – N.I. a2, d32, b3. Effect 7 – Trauma – > Sensation seeking – > Addiction – N.I. a3, b3. Direct effect – X on Y including the influence of M1, M2 and M3 – c‘.

**FIGURE 4:**
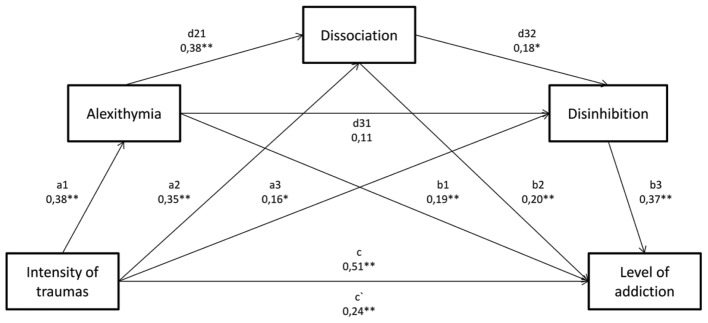
The model of mediation for the impact of traumatic experiences’ intensity on the inclination toward alcohol addiction, with three mediators, i.e.: alexithymia, dissociation, and disinhibition. Below, paths were described, which were calculated in the model. ^∗^*p* < 0.05; ^∗∗^*p* < 0.01. Direct effect – X on Y without the influence of M1, M2 or M3 – c. Effect 1 – Trauma – > Alexithymia – > Addiction – significant a1, b1. Effect 2 – Trauma – > Alexithymia – > Dissociation – > Addiction – significant a1, d21, b2. Effect 3 – Trauma – > Alexithymia – > Disinhibition – > Addiction – N.I. a1, d31, b3. Effect 4 – Trauma – > Alexithymia – > Dissociation – > Disinhibition – > Addiction – significant a1, d21, d32, b3. Effect 5 – Trauma – > Dissociation – > Addiction – significant a2, b2. Effect 6 – Trauma – > Dissociation – > Disinhibition – > Addiction – significant a2, d32, b3. Effect 7 – Trauma – > Disinhibition – > Addiction – significant a3, b3. Direct effect – X on Y including the influence of M1, M2 and M3 – c.

The obtained results provided the basis for constructing two subsequent mediation models with three mediators (**Figures 3**, **4**). In the first model, the intensity of traumatic experiences were introduced as the main independent variable, and alexithymia, dissociation, and sensation seeking constituted the mediating variables. The main dependent variable was the intensification of alcoholic behaviors. With a non-standard macro for Hayes’ SPSS Process (2013) the mediation analysis was carried out with three mediators (model 6), which included alexithymia, dissociation, and sensation seeking. The sequence of introducing mediators to the model resulted from the previous analysis. The assumed model with three mediators turned out well fitted to data and statistically significant *F*(4,196) = 35.1964; *p* < 0.001. In order to understand the mechanism related to the development of an inclination toward addiction, the analysis of particular intermediary effects was interesting. Effect 1, 2, and 5 proved to be statistically important. The first significant effect relates to the connection between the intensity of traumatic experiences and alexithymia, and the intensification of alcoholic behaviors (bootstrap confidence interval < 0.416; 0.1583 >). The greater intensity of traumas, the higher level of alexithymia (*a*1 = 0.38; *p* < 0.01), which, as a consequence, increases the risk of addiction (*b*1 = 0.24; *p* < 0.01). The other important indirect effect (bootstrap confidence interval < 0.0127;0.0741 >), which takes dissociation into account, indicates its significance with alexithymia (*d*21 = 0.38; *p* < 0.01), and alexithymia and dissociation materially influence the growth of a tendency toward addiction (*b*2 = 0.25; *p* < 0.01).

The last significant indirect effect 5 (bootstrap confidence interval < 0.378;0.1606 >) shows the impact of traumatic experiences and dissociation (*b*2 = 0.25; *p* < 0.01) on a tendency toward addiction. Summarizing the analysis of the discussed model, it may be stated that alexithymia and dissociation both, separately, i.e., as independent mediators, as well as in the doubled mediation, increase the intensification of alcoholic behaviors. In the tested model, the temperament was taken into account in line with theoretical assumptions, however, its impact proved to be immaterial (**Figure 3**). Due to this fact, it was decided that in a subsequent mediation analysis, one of temperament dimensions would be taken into account, i.e., disinhibition, as the analysis of correlation matrix (**Table 1**) indicated moderate correlation between disinhibition and addiction. The described model is statistically significant *F*(4,196) = 52.1747; *p* < 0.001. Compared to the previous model, apart from effect 1, 2, and 5, also the mediation impact of disinhibition on addiction (effect 7) turned out significant, as well as the double mediation with dissociation (effect 6), and effect 4 combining all mediators, which were found to influence significantly the dependency between the intensity of traumatic experiences and addiction.

**Table 1:** The matrix of correlations between the level of alexithymia, dissociation, and sensation seeking, and an addiction to alcohol.

	Level of alcohol addiction
	*Pearson’s r*
Alexithymia – general score	*0.483^∗∗^*
Difficulty in verbalizing emotions	*0.442^∗∗^*
Difficulty in identifying emotions	*0.440^∗∗^*
Operational style of thinking	*0.368^∗∗^*
Dissociation – general score	*0.533^∗∗^*
Amnesia level	*0.545^∗∗^*
Absorption level	*0.413^∗∗^*
Depersonalization level	*0.497^∗∗^*
Sensation seeking – general score	*0.218^∗∗^*
Thrill and adventure seeking	*0.058*
Experience seeking	*-0.047*
Disinhibition	*0.544^∗∗^*
Boredom susceptibility	*-0.044*


*^∗^p < 0.05; ^∗∗^p < 0.001.*

The presented model is a partial repetition of the previous one, hence important effects, which differentiate both models, will be discussed. Beginning with an indirect effect 7 (bootstrap confidence intervals < 0.0063;0.1186 >), which combines the dependency between the intensity of traumatic experiences and disinhibition (*a*3 = 0.16; *p* < 0.05), as well as disinhibition and the intensification of alcoholic behaviors (*b*3 = 0.37; *p* < 0.01). Another important indirect effect is effect 6 (bootstrap confidence intervals < 0.0045;0.0563 >), which extends indirect effect 7 by dissociation, which relates materially to disinhibition (*d*32 = 0.18; *p* < 0.05). The most important significant indirect effect in the discussed model is effect 4 (bootstrap confidence intervals < 0.0017;0.0249 >) which combines all mediators, and justifies the impact of all factors jointly on the intensification of alcoholic behaviors (a1, d21, d32, b3). Summarizing the obtained results, it may be assumed that all, i.e.: alexithymia, dissociation, as well as disinhibition are important mediators which influence the relation between the intensity of traumatic experiences and alcohol addiction (**Figure 4**).

## Discussion

The purpose of the study was to verify the model related to the connection of traumatic experiences’ intensity with account given to factors pointed out by other scholars as significant for the affective regulation disorders manifested, among others, as an addiction to alcohol. As a result of performed analysis, the relation was fully confirmed between trauma and addiction (a direct effect), which takes into account indirect effects of alexithymia, dissociation, and disinhibition in the development intensification of alcoholic behaviors. Traumatic experiences are largely connected with the occurrence of dissociation symptoms, which is confirmed in numerous reports (Carlson et al., 2009; Liotti and Farina, 2016). A tendency to isolate traumatic memories explains reaching for alcohol in difficult situations; hence binge drinking, which is called a chemical dissociation, constitutes one of possible consequences of traumatic experiences’ volume (Klanecky et al., 2008). The absence of capability to name and modulate the affect consciously, by heightening tensions and negative emotions, results in relieving agitation with alcohol. With account given to the connection of alexithymia with anxiety-related attachment styles, it is to be expected that the absence of protection measures in the form of developed accurate strategies of self-regulation will constitute a particular risk factor of reaching for alcohol in response to stress (Evren et al., 2008, 2009; Craparo et al., 2014; Zdankiewicz-Ścigała, 2017). The performed analyses confirmed the hypothesis regarding the mediation role of dissociation in the relation between traumatic experiences and an inclination toward alcohol addiction. Dissociation aims to protect the Self at every stage of development by introducing changes within consciousness. It operates through the process of inhibition, which prevents distressing information to enter the consciousness due to the construction of a parallel, more favorable reality, in which an individual is able to find a shelter (Carlson et al., 2009).

The relief achieved due to the withdrawal to a mental shelter for certain time (Steiner, 1993) is not pathological, and it may be used to the service of the Self, to protect powers, creativity, and relations with others. However, if the withdrawal leads to losing control over agitation, a strong desire to isolate and distort the Self and relations with others may occur. Something, which was supposed to protect and secure against suffering, freezes causing a strong dissonance until the loss of primary contact with reality in favor of self-appeasement and addiction (Schimmenti and Caretti, 2010).

The last verified mediation referred to the relation between the need for stimulation and the level of alcohol addiction, with the simultaneous impact of alexithymia and dissociation. Along with the growth of disinhibition feature on the scale of sensation seeking, the inclination toward alcohol addiction is increased. The result is in line with the studies quoted above and relating to this dimension of sensation seeking. The inclination toward alcohol addiction is connected both with the consequences of traumatic development and the intensity of traumas, as well as biological factors, which, through the demand for stimulation, may determine the direction for the selection of dealing strategies. An inclination toward the pathological dissociation and blocking the understanding and experiencing of emotions in the long term result in the creation of poor mental representation of emotions (Taylor et al., 1991; Maruszewski and Ścigała, 1998). It may be concluded that it affects disorders and deficits related to the mentalization of emotional states (Allen et al., 2014). As a matter of fact, mentalization deficits block dealing with life adversities based on the reflection and self-control, particularly in difficult, new, and stressful conditions. What is more, this causes disturbances in building and executing life plans. In this sense, the inclination toward addictions is to be understood as the symptom of dealing with persistent emotional suffering. The addiction is a consequence of an individual’s disorders, i.e., the emotional immaturity, and alcohol may constitute a measure to deal with difficult situations (Cierpiałkowska, 2010). In the conditions of severe stress, the right hemisphere of the brain may lose its capacity to integrate between cortical structures and subcortical structures. Then, the limbic and autonomous information is processed exclusively on the lower level of a right amygdala, and the blockade is formed against its access to higher neural structures (i.e., anterior callosal gyrus and orbitofrontal areas) (Schore, 2009). Numerous studies have also documented dysfunctions observable in the brain activity in the processing of emotogenic stimuli in persons with the high level of alexithymia. It was proven that the cognitive dimension of alexithymia, and in particular, the aspect of identifying and diversifying emotions, is related to the increased activity of posterior insula, as well as anterior and central cingulate cortex. It is also related to the decreased activity of prefrontal cortex, amygdala, and anterior insula. On the other hand, in the affective dimension, the low emotional excitability is related to a greater volume of central cingulate cortex, and the limited imaginational capabilities are related to a decreased activity of posterior cingulate cortex (Moriguchi et al., 2007; van der Velde et al., 2013). The childhood experiences, which are harmful to the development, may leave the permanent physiological reactivity in the limbic areas of the brain (Post, 1992). The emotional and social deprivation distorts the normal development of synaptic structures, and leads to neurological defects which constitute the basis for subsequent behavioral and cognitive deficits (Poeggel and Braun, 1996; Poeggel et al., 1999). Under the extreme stress, both hyperactivation and hypoactivation of cortical parts of the right hemisphere cause the loss of the capability to integrate sensory processes. Early age unfavorable experiences cause the growth of sensitivity to later stressful events, and result in the increase of individual susceptibility to the development of psychiatric disorders (Graham et al., 1999).

Dissociation may interfere with the connections between affects, cognitions, and voluntary behavior control by influencing the development of alexithymia and resulting in the dissociation of the physiological, cognitive, and affective components of emotions. Both, dissociation and alexithymia have been considered impairments of emotive perception that help trauma survivors manage overwhelming or difficult affective states. Trauma, alexithymia, and dissociation are interrelated among individuals with alcoholism. This study confirmed the existing research regarding the role of trauma in addiction behavior. Alcohol dependency can be considered a dissociative reaction of individuals with difficulties in identifying, expressing, and regulating emotions. This triad’s relevance for the prevention and treatment of alcohol dependence requires attention in future studies. The current results suggest that addictive behaviors have a dissociative nature that allows individuals to manage negative and unregulated emotions (Craparo et al., 2014; Zdankiewicz-Ścigała, 2017). The present study measured chronic (trait) dissociation rather than acute and transient dissociative states and alexithymia as personality traits. Although chronic dissociation contributes to difficulties in identifying feelings, chemically (alcohol-induced) transient dissociative states may be a paradoxical effort to identify and express feelings that are otherwise difficult to access. Thus, alcohol appears to influence the interplay between trauma, dissociation, and alexithymia. This interaction may be an important factor in the prevention and treatment of alcoholism in the addicted population.

## Conclusion

Alexithymia and dissociation may be considered as defense mechanisms, which form the traumatic development (Liotti and Farina, 2016). The disintegration in the personality development, which continues from an early childhood, involves the following levels: neurobiological, mental, and social one. The dissociation of mental processes relates, among others, to the split of consciousness stream in threatening and possibly threatening situations, which contributes to permanent personality disorders (van der Hart et al., 2006). Alexithymia, on the other hand, by distorting appropriate recognition and understanding of emotions, affects the inappropriate use of emotions as important processes that inform of the individual’s own mental condition, and of relations with other people, i.e., it is responsible for mentalization deficits. As a consequence, it leads to dysfunctions in processes responsible for regulating emotions. Taken together this study has confirmed important relationships between early trauma, alexithymia, dissociation, and addition.

## Limitations

When indicating the limitations of the conducted study, it should be assumed that the sensation seeking is a temperament feature, which, as is shown by many studies, is weakened with age, and the examined sample was characterized by a very high age variance, and substantially lower mean age in the control group than in a group of alcohol addicts, despite in the group of addicts, scores in the temperament test were substantially higher. The greatest difficulty and limitation was the selection of subjects to homogeneous groups as regards age and gender. An ideal age criterion for investigating interactions between the temperament and the consequences of traumatic development would be an early adulthood. The time relates to the insufficiently developed mechanisms of modulating emotions with the simultaneous escalation of risky behaviors. Examining addicted individuals during this type of development asymmetry (McCabe et al., 2015) with the simultaneous comparison to a control group (non-addicted individuals), would enable showing full interactions of mentioned factors in sensation seeking. The conducted research is a correlation type of study. In the context of trauma experience, it is difficult to imagine the experimental research conducted ethically in this area. In this type of studies, the range of examined variance is crucial. The scores obtained in our research are significant, but the variance under explanation is moderate. It means that in subsequent studies, it is necessary to take account of other factors, which might contribute to the fact that in regulating affect people resort to stimulants, including alcohol. In addition, the subjects, who agreed to participate in the research, are addicted individuals who are in voluntary treatment, and who fill in questionnaires voluntarily. Hence, the sample is undoubtedly of a biased nature. Based on the above, it may be stated that diagnosed alcohol addicts have faced traumatic experiences, and individuals characterized by the high level of dispositional alexithymia and dissociation treat alcohol as a way to relieve affective tension. Alcohol is therefore a form of chemical dissociation in their case. The generalization of results obtained from sample subjects, who suffered trauma in early childhood, and who have a high level of alexithymia and dissociation, is ineligible. Although many research results indicate predictions toward mental disorders and mental illnesses in individuals who experienced chronic trauma in childhood.

## Author Contributions

EZ-Ś conceived and designed the work. DŚ acquired and analyzed the data. EZ-Ś and DŚ interpreted the data, drafted the work and revised it critically for important intellectual content, approved the final version of the manuscript to be published, and agreed to be accountable for all aspects of the work in ensuring that questions related to the accuracy or integrity of any part of the work are appropriately investigated and resolved.

## Conflict of Interest Statement

The authors declare that the research was conducted in the absence of any commercial or financial relationships that could be construed as a potential conflict of interest.
